# The relationship of lactating beef cow metabolizable energy intake to energy partitioning, milk composition, and calf performance

**DOI:** 10.1093/jas/skaf126

**Published:** 2025-04-21

**Authors:** Courtney M Williams, Corbit L Bayliff, Mariana E Garcia-Ascolani, Ryan R Reuter, Gerald W Horn, Carla L Goad, David L Lalman

**Affiliations:** Livestock Nutrition Center, Overland Park, KS 66210, USA; Park City, KS 67219, USA; Department of Animal and Food Sciences, Oklahoma State University, Stillwater, OK 74078, USA; Department of Animal and Food Sciences, Oklahoma State University, Stillwater, OK 74078, USA; Department of Animal and Food Sciences, Oklahoma State University, Stillwater, OK 74078, USA; Department of Statistics, Oklahoma State University, Stillwater, OK 74078, USA; Department of Animal and Food Sciences, Oklahoma State University, Stillwater, OK 74078, USA

**Keywords:** energy partition, metabolizable energy, beef cows, Angus, milk, body composition

## Abstract

A 2-yr experiment was conducted to determine the impact of maternal metabolizable energy intake (**MEI**) on energy partitioning and performance of beef cows and calves. Forty mature crossbred Angus beef cows (6 ± 2.0 yr, 534 ± 60 kg BW) were used each year along with their suckling steer calves (84 ± 8.7 d, 130 ± 15 kg BW). Cows were stratified by early lactation milk yield and randomly assigned to 1 of 5 levels of MEI. Each treatment group was housed in a dry lot pen. During year 1, maternal MEI ranged from 225 to 320 kcal·BW^0.75^·d^−1^, while in year 2, MEI ranged from 215 to 288 kcal·BW^0.75^·d^−1^. Calves were provided ad libitum access to the same TMR diet as the cows in a creep area. Parameters evaluated included apparent total tract nutrient digestibility, milk production and nutritional composition, cow body weight (**BW**), body condition score (**BCS**), ultrasound carcass characteristics, calf BW, and calf creep feed intake. Data were analyzed using the GLIMMIX procedure of SAS (SAS Inst. Inc., Cary, NC) as a general linear mixed model. The final model included the linear effect of maternal MEI as a fixed effect and the year of the experiment as a random effect. Significance of the fixed effects was declared when *P* ≤ 0.05, while tendencies were declared when 0.05 < *P* ≤ 0.10. Cow 100-d BW, BCS, and ADG linearly increased (all, *P *< 0.01) with increasing levels of maternal MEI. Similarly, calf 100-d BW and ADG linearly increased (*P* = 0.03, *P* < 0.01, respectively) with increasing maternal MEI. Milk yield, milk energy production and all milk nutrients linearly increased (*P *≤ 0.04) with increasing maternal MEI, except for milk urea nitrogen which significantly decreased (*P *< 0.01). Cow energy partitioned to tissue accretion and milk production linearly increased (both, *P* < 0.001). As maternal MEI increased, the proportion of net retained energy partitioned to maternal tissue accretion initially increased. However, at approximately 275 kcal·BW^0.75^·d^−1^ of maternal MEI, the proportion plateaued. Increased maternal MEI reduced the efficiency of calf BW gain (BW gain per unit of calf MEI from both creep feed and milk) in a linear fashion (*P *= 0.03). These findings suggest that maternal energy intake, rather than genetic capacity for milk yield, limited milk energy production in these cows. Furthermore, the maximum proportion of retained energy as maternal tissue was achieved at about 275 kcal·BW^0.75^·d^−1^.

## Teaser

The current Beef Cattle Nutrient Requirements Model (NASEM, 2016) does not provide an adjustment for milk yield or milk nutrient concentration over a range of maternal energy supply. In this experiment, energy was simultaneously partitioned to maternal tissue and milk energy in a linear fashion. These relationships were characterized by regression models.

## Introduction

Characterization of production responses to feed energy intake is a critical element in designing beef production systems that optimize the efficiency of grazing resources or harvested feed utilization. In the lactating beef cow, available nutrients are partitioned to maintenance, milk synthesis, maternal tissue, and later in lactation, to pregnancy (Jenkins and Ferrell, 1992). Multiple energy sinks increase complexity in nutritional modeling because the response to increasing energy may be limited and (or) may not be linear. The current Beef Cattle Nutrient Requirements Model (NASEM, 2016) does not provide an adjustment for milk yield or milk nutrient concentration when maternal energy supply is increased. Previous work has shown that increasing energy intake increases total milk yield in beef (Jenkins and Ferrell, 1992; Jenkins et al., 2000; Miller et al., 1999; Lalman et al., 2000) and dairy cows (Macleod et al., 1984; Coulon and Rémond, 1991; Friggens et al., 1995; Broderick, 2003). Increasing feed energy intake increases milk energy concentration and daily milk energy output in beef (Lalman et al., 2000; Winterholler et al., 2012) and dairy cows (Coulon and Remond et al., 1991; Friggens et al., 1995; Broderick, 2003). Clearly, milk yield and milk energy production can be manipulated by energy intake in the beef cow. However, it can also be limited by the genetic capacity for milk yield (Jenkins and Ferrell, 1992). The work of  Jenkins and Ferrell (1992) documented a significant influence of breed and energy intake level on the time of peak lactation, yield at peak lactation, and total 210-d milk yield. In this work, daily energy intake was estimated using tabular feed energy values multiplied by dry matter intake. However, diet digestibility is influenced by the level of feed intake in lactating beef cows (Trubenbach et al., 2014). In many situations, energy and protein supplementation may be intended to maintain or gain cow body condition score (**BCS**; Wagner et al., 1988) rather than maximize milk yield or milk energy yield. Little data is available to characterize the partitioning of retained energy with changes in metabolizable energy intake (**MEI**) (Lalman et al., 2000; Freetly et al., 2006). The objective of this experiment was to characterize the dynamic relationship of MEI in lactating beef cows to energy partitioning, milk yield, milk composition, and performance of nursing calves.

## Materials and Methods

The experiment was conducted at the Oklahoma State University Range Cow Research Center, located near Stillwater, Oklahoma. The Oklahoma State University Institutional and Animal Care and Use Committee approved all procedures involving the use of animals (IACUC Protocol number OKL02911).

### Animals and facilities

Eighty lactating beef cows (≥ 87.5% Angus, 6 ± 2.0 yr, 534 ± 60 kg body weight [**BW**]) and their steer calves (84 ± 8.7 d, 130 ± 15 kg BW) were used in a 2-yr experiment (40 cow/calf pairs per year). All calves and their dams were sired by Angus bulls. The average calf birth date was March 15 in yr1 and March 8 in year 2. Steers were castrated at birth by the application of a rubber ring (Chase et al., 1995) and received an anabolic implant (Ralgro, Merck Animal Health, Madison, NJ) at approximately 2 mo of age. Cows were stratified by early lactation milk yield and then randomly assigned within stratification group to 1 of 5 outdoor dirt-floor pens in groups of 8. Groups were rotated among pens every 28 d to minimize the effect of pen. Each pen provided 89 m^2^ per cow–calf pair and was fitted with concrete, fence-line feed bunks to provide 0.9 m of linear bunk space per cow and 0.3 m of linear bunk space per calf in a creep area. Cows and calves had access to 4.2 m^2^ per cow of shade and each pen was equipped with an automatic waterer.

### Experimental diets and feeding management

Diet ingredient components and chemical composition are shown in Table 1. A range of cow energy intake was created by varying the daily feeding rate of the same diet (within year; Table 2). The average values of DMI per group and year, as well as the maternal MEI are presented in Table 3. During year 1, maternal MEI ranged from 225 to 320 kcal·BW^0.75^·d^−1^, while in year 2, these values ranged from 215 to 288 kcal·BW^0.75^·d^−1^. A vitamin and mineral supplement (11.7% Ca, 10.29% P, 1.2% Mg, 1,047 mg/kg Cu, and 7,631 mg/kg Fe) was top-dressed on both cow feed and calf feed at the rate of 57 g^−1^ ∙ d^−1^ in year 1. Vitamins and trace minerals were included in the liquid supplement in year 2. To ensure that neither rumen degradable protein (**RDP**) nor metabolizable protein (**MP**) were limited at each level of feed intake, Model level 1 (NRC, 2000) was used to estimate RDP and MP balance using microbial efficiency = 12% of TDN intake (NRC, 2000). Before the initiation of the experiment in year 1, MP balance was estimated to be slightly negative. Therefore, 0.23 kg of cottonseed meal was top dressed on cow feed for the lowest feed intake group. All other treatment groups were projected to receive adequate (positive balance) RDP and MP without additional protein supplementation.

**Table 1.** Total mixed ration formulation and diet nutritional composition across experiment year

	Year 1	Year 2
Ingredient, % DM		
Corn gluten feed^1^	54.8	—
Prairie hay, chopped	30	—
Corn, cracked	12.7	21
Limestone, 38%	2.5	2
Bermudagrass hay, chopped	—	37.5
Distillers grain	—	29
Liquid supplement^2^	—	7.5
Soybean meal	—	2.5
Salt	—	0.5
Nutritional composition, % DM		
DM, % as fed	72.7	88.4
TDN, %^3^	67.2	66.7
ME, Mcal/kg^4^	2.46	2.45
NEm, Mcal/kg^5^	1.56	1.57
CP, %	14.7	15.6
EE, %	3.7	3.9
ADF, %	27.3	27
NDF, %	52.9	47.7
Ash, %	7.99	7.64

^1^Sweet Bran (Cargill, Inc., Minneapolis, MN).

^2^The liquid supplement contained 1.76 % Ca, 1.85% P, 1.25% Mg, 916.74 ppm Cu, and 527.75 ppm Fe per kilogram DM (Quality Liquid Feeds Inc., Dodgeville, WI).

^3^TDN = truly digestible non-fiber CHO + in vitro truly digestible NDF + truly digestible crude protein + ((EE − 1) × 2.25) − 7 (NRC, 2001).

^4^ME = DE × 0.82 and DE = (truly digestible non-fiber carbohydrate/100) × 4.2 + (truly digestible NDF/100) × 4.2 + (truly digestible crude protein/100) × 5.6 + ((EE − 1)/100) × 9.4 − 0.3 (NRC, 2001).

^5^NEM = 1.37 ME − 0.138 × ME^2^ + 0.0105 × ME^3^ − 1.12 (NRC, 2000).

**Table 2.** Daily feeding rate and maternal MEI^1^ of lactating Angus cows during mid to late lactation across experiment year

	DMI^2^, kg DM·d^−1^	DMI, g. kg (BW^0.75^)^−1^ ∙ d^−1^	MEI, kcal·(BW^0.75^)^−1^ ∙ d^−1^
Year 1	8.7	85	225
	10.8	100	255
	12.5	111	274
	14.1	126	299
	15.2	140	320
Year 2	8.6	81	215
	9.5	89	233
	10.5	99	253
	11.4	108	269
	12.6	119	288

^1^Metabolizable energy intake = [ME of the feed (kcal) × DMI (kg)] / BW^0.75^ (kg) was calculated from regressing in vivo digestible energy values on DMI (g/kg BW^0.75^). Feed digestible energy was converted to metabolizable energy by multiplying by 0.82 (NASEM, 2016).

^2^Dry matter intake.

**Table 3.** Summary of production parameters for cows and calves under varying maternal MEI^1^ across year of experiment

	Year 1				Year 2			
	Min^2^	Mean	Max^3^	SD^4^	Min	Mean	Max	SD
Cow								
BW^5^ 0 d, kg	488	527.2	555	26.2	501	506.6	512	4.4
BW 100 d, kg	497	564	599	42.2	520	534	555	13.8
ADG^6^, kg	0.1	0.37	0.62	0.209	0.13	0.27	0.42	0.104
BCS^7^ 0 d, kg	5.1	5.3	5.5	0.18	3.6	4	4.3	0.24
BCS 100 d, kg	4.4	5.4	6.1	0.69	3.6	4.3	5.3	0.7
DE^8^, kcal·kg^−1^ DMI	2,720	3,012	3,260	197.2	2,850	3,104	3,350	184.6
DM^9^ Digestibility, %	66.9	75.4	84.9	7.07	71	76.7	84.4	5.34
Tissue RE^10^, *NE_t_* kcal·(BW^0.75^)^−1^	−7.9	12.8	29.7	15.14	−2.4	10.1	21.6	11.5
Milk RE, *NE_l_* kcal·(BW^0.75^)^−1^	47	64.5	83.1	13.44	46.3	59	67	8.5
Total RE^11^, *NEr* kcal·(BW^0.75^)^−1^	43.7	80.7	115.7	27.73	47.8	72.2	91.3	18.8
REA^12^, cm^2^	55.4	66.7	74.0	7.10	60.6	64.3	68.3	3.60
IMF^13^, %	3.7	4.2	4.4	0.29	3.6	3.8	4.2	0.26
Rib fat, cm	0.2	0.4	0.5	0.13	0.2	0.3	0.3	0.07
Rump fat, cm	0.2	0.4	0.6	0.19	0.2	0.3	0.3	0.07
Calf								
BW 100 d, kg	257	274.2	284	10.2	258	269	279	7.7
ADG, kg	1.26	1.38	1.45	0.074	1.4	1.44	1.5	0.043
DMI^14^, kg·d^−1^	3.9	4.1	4.3	0.15	3.9	4.3	4.6	0.24
DM digestibility, %	68	71.7	75.9	2.82	65	69.3	73.7	3.42
Creep feed MEI^15^, kcal MEI·(BW^0.75^)^−1^·d^−1^	183	195	203	7.8	195	204	217	9.5
Milk MEI, kcal MEI·(BW^0.75^)^−1^·d^−1^	151	206	258	39.8	127	174	202	29.5
Efficiency, g ADG·(Mcal MEI)^−1^	59.5	63.6	69.2	3.53	68.4	72.4	76.8	3.96
REA, cm^2^	62.7	64.5	67.5	2.16	64.7	66.5	68.7	1.76
IMF, %	3.3	3.4	3.7	0.18	3.1	3.3	3.6	0.15
Back fat, cm	0.5	0.5	0.6	0.03	0.5	0.5	0.6	0.05
Rump fat, cm	0.5	0.6	0.6	0.06	0.6	0.6	0.7	0.04
Milk								
Fat, %	3.8	4	4.2	0.17	2.9	3.2	3.7	0.3
Protein, %	2.9	3.1	3.2	0.12	3	3.1	3.2	0.1
Lactose, %	4.8	4.9	4.9	0.04	4.8	4.8	5	0.08
SNF^16^, %	8.7	8.9	9	0.15	8.8	8.9	9.2	0.17
MUN^17^, %	11.8	12.7	13.7	0.88	18.9	19.3	19.9	0.48
MEP^18^, Mcal·kg^−1^	0.74	0.77	0.79	0.023	0.66	0.69	0.75	0.036
Milk yield, kg·d^−1^	6.9	9.5	11.8	1.79	6.4	8.5	9.9	1.31

^1^Metabolizable energy intake = [ME of the feed (kcal) × DMI (kg)] / BW^0.75^ (kg) was calculated from regressing in vivo digestible energy values on DMI (g/kg BW^0.75^). Feed digestible energy was converted to metabolizable energy by multiplying by 0.82 (NASEM, 2016).

^2^Minimum value.

^3^Maximum value.

^4^Standard deviation.

^5^Body weight.

^6^Average daily gain.

^7^Body condition score.

^8^Digestible energy.

^9^Dry matter.

^10^Tissue retained energy.

^11^Total net energy retained.

^12^Rib eye area.

^13^Intramuscular fat.

^14^Dry matter intake.

^15^Metabolizable energy intake from creep feed.

^16^Solids nonfat.

^17^Milk-urea nitrogen.

^18^Milk energy production.

Cows were acclimated to the experimental diet for 19 d in year 1 and 23 d in year 2. Cows and calves were managed as a group during the first 6 d of adaptation while grazing bermudagrass pasture and simultaneously increasing the mixed diet feeding level at the rate of 2 kg increase every second day. Cows were sorted into their respective groups and moved into the pens on day −13 (year 1) and day −17 (year 2) while mixed diet intake level adjustment continued at the rate of 2 kg every other day (maximum) until each group’s targeted intake level was reached (Table 2). Experimental treatments began (day 0) on June 18 and June 13 and continued for 97 d and 108 d in year 1 and year 2, respectively. Feeding occurred at approximately 0730 hours each day. Prior to feed delivery, calves were gathered and enclosed in the shaded areas and remained separated until the cows consumed their ration. This ensured that calves did not have access to cow feed. Calves had unrestricted access to the creep area through a creep gate, which the cows could not access. Calves received the same diet as the cows and daily amounts provided were increased as needed to ensure ad libitum intake. Orts from the creep areas were removed weekly during year 1 and daily in year 2 and sampled for analysis of nutritional composition weekly both years. No orts were recorded from the cow feed bunks during either year, since cows consumed all feed that was offered. Individual BCS of the cows was assigned independently by 2 experienced technicians every 28 d and the average value was computed in the data analysis.

### Apparent total tract nutrient digestibility

Apparent total tract nutrient digestibility was determined using acid detergent insoluble ash (**ADIA**) as the internal marker (Cochran and Galyean, 1994; Kanani et al., 2014). Representative feed samples were collected from the feed bunks each morning from days 90 to 95 and days 68 to 73 in year 1 and year 2, respectively. Fresh manure samples were collected from the pen surface or from rectal grab samples from cows (n ≥ 5) and calves (n ≥ 5) each morning and night (12 h apart) on days 69 to 74 and days 91 to 96. Samples were only collected from the pen surface if defecation was observed and the sample could be collected immediately, taking care to avoid contamination from soil particles. Orts from the calf bunks were collected prior to feeding on the days of manure collection. Feed samples from each collection were placed in paper bags and dried in a forced air oven (50 °C; 52-h minimum). Manure samples were frozen immediately (−80 °C) and placed in a freeze dryer later (Virtis 213521, SP Scientific, Gardiner, NY) until all the moisture was extracted. Feed and manure samples were then ground using a Wiley Mill (Thomas Scientific, Swedesboro, NJ) using a 1-mm screen. Equal amounts of ground samples from each day were pooled within treatment. Acid detergent fiber (**ADF**) was determined using an ANKOM 2000 Automated Fiber Analyzer (ANKOM Technology, Macedon, NY) according to the manufacturer’s protocols. After determination of ADF, filter bags containing the samples were ashed (500 °C; 8 h) to obtain ADIA.

### Animal health

Prior to experiment initiation in year 1, the steer calves received a clostridial vaccine (Covexin 8, Merck Animal Health, Madison, NJ) and pour-on dewormer (Normectin, Norbrook Inc., Lenexa, KS). At experiment initiation in year 1, cows and calves were treated with an insecticide (Synergized Permethrin, Durvet, Inc., Blue Springs, MO) and drenched with a dewormer (Valbazen, Zoetis Inc., Florham Park, NJ). On day 26, cows were given another dose of pour-on Permethrin for fly control. Approximately 1 mo later, the cows received insecticide ear tags (XP820, Y-Tex Corporation, Cody, WY). One week prior to weaning, steers were revaccinated with Covexin 8 and BoviShield Gold 5 (Zoetis Inc., Florham Park, NJ). On day 0 in year 2, all steers were administered a clostridial (Vision 7, Merck Animal Health, Madison, NJ) and respiratory vaccine (Titanium 5, Elanco Animal Health, Greenfield, IN) as well as administered a dewormer (Safeguard, Merck Animal Health, Madison, NJ). At this same time point, all cows were administered Safeguard and XP820 insecticide ear tags. On the last day of the experiment (day 125), steers were revaccinated with Titanium 5 and Vision 7.

### Cow breeding protocol

Prior to the experiment, all cows were synchronized for artificial insemination (**AI**) using a Co-Synch protocol (Stein et al., 2015). A controlled internal drug release (**CIDR**; Zoetis Inc., Parisppany, NJ) device containing progesterone was inserted into the vagina and Factrel (gonadorelin hydrochloride, Zoetis Inc., Parisppany, NJ) was injected intramuscularly (IM). After 7 d, the CIDR was removed and Lutalyse (dinoprost tromethamine, Zoetis Inc., Parisppany, NJ) was given IM. Approximately 60 h later AI was performed and a second Factrel injection was administered. Following AI, cows in year 1 were monitored by Heatwatch Estrus Detection System (CowChips, LLC, Manalapan, NJ) for 45 d. If estrus was detected, the cow was artificially inseminated 12 h after observation of standing heat. In year 2, blood samples were taken from the coccygeal vein 20 d after AI to determine pregnancy status. Heat detection patches (Estrotect, Estrotect Inc., Spring Valley, WI) were applied to those cows determined to be open. Morning and night visual heat checks were performed on those with heat detection patches. Cows were artificially inseminated 12 h following observation of standing heat. Palpation pregnancy checks were performed on December 10 in year 1 and October 26 in year 2.

### Milk yield and composition

Milk yield was measured 28 and 20 d prior to experiment initiation in years 1 and 2, respectively, to be used for treatment allotment. After experiment initiation, milk yield was determined every 28-d using a milking procedure adapted from Marston et al. (1992). All cows were milked using a portable milking machine (Portable Vacuum Systems, Springville, UT). On the day prior to milking, calves were separated from their dams at 1400 hours. During this time, they were allowed access to water but did not have access to creep feed. Calves were reunited with their dams at 2000 hours (6-h interval) for a 45-min nurse out period. Calves were separated again at 2045 hours from their dams, and milking began at 0500 hours (8.25-h interval) the following morning, resulting in an average separation time of 8 h. On the morning of milking, cows were combined into 1 pen and brought into the working facility in random order. After entering the working facility, cows were weighed on a calibrated scale (Sooner Scale Inc., Oklahoma City, OK) and sent to 1 of 2 working chutes, allowing 2 cows to be milked simultaneously. Once in the chute, cows were injected IM with 1 ml oxytocin (Oxoject, Henry Schein Animal Health, Dublin, OH) for milk let down. Udders were then washed with warm, soapy water, dipped with an antibacterial solution, dried, and hand-stripped before application of the milking claw. The milking claw remained on the udder until milk flow ceased. Each quarter was then hand-stripped to ensure complete udder evacuation. At the completion of milking, teats were again dipped with the antibacterial solution and the cow was returned to her calf. Any hand-stripped milk obtained was combined with the machine milk and weighed on a calibrated platform scale (Defender 5000, Ohaus Corp., Parsippany, New Jersey). A subsample was taken in a vial containing 2-bromo-2nitropropane-1,3-diol for preservation and shipped to the Heart of America Dairy Herd Improvement Association laboratory (Manhattan, KS) for composition analysis. Milk energy content of each sample was estimated utilizing the following equations (equations 4 to 17, NRC, 2000):


$$\begin{array}{l} E=\left(0.092\;\times \%MkFat\right)+\left(0.049\;\times \%MkSNF\right)-0.0569 \end{array}$$

Where *E* is milk energy content (Mcal/kg of milk), MkFat is concentration of fat in milk, and *MkSNF* is concentration of solids nonfat in milk. Actual time of milking and milk yield was recorded for each cow. Milk yield was adjusted to a constant 8-h separation time and subsequently multiplied by 3 to represent 24-h milk yield.

### Retained energy calculations

Daily net energy partitioned into milk (NE_l_, Mcal·d) was calculated as:


$$\begin{array}{l} NE_{l}=Yn\times E\; \end{array}$$

Where *NE*_*l*_ is net energy of lactation, *Yn* is daily milk yield, and *E* is milk energy content.

The average of the 4 NE_l_ estimates was used to determine daily net energy partitioned to milk production. Calf NE_l_ intake was considered to be equal to cow NE_l_ production.

Maternal tissue retained energy (NE_t_, Mcal·d) was calculated using the Beef Cattle Nutrient Requirements Model (NASEM, 2016). Briefly, equations first published by NRC (1996, 2000) use BCS to compute the proportion of empty BW that is fat and protein. Next, body protein and fat proportion are multiplied by empty BW to determine total body fat and total body protein at days 0 and 100 of the experiment. Finally, at each timepoint, total body fat (kg) and total body protein (kg) are multiplied by their biological energy value (9.4 and 5.7 Mcal/kg, respectively). Calculated total body energy for day 0 was subtracted from calculated total body energy for day 100 to determine tissue net energy change (Mcal NE_t_). If BW loss occurred during the experimental phase, the loss in energy was multiplied by 0.8 to estimate Mcal NE_t_ available (NASEM, 2016). Net energy required for pregnancy (NE_y_, Mcal/d) was calculated retrospectively using average calf birth BW and calf birth date from the subsequent calving season as follows (NASEM, 2016):


$$\begin{array}{l} NEy=\left[CBW\times \left(0.5855-0.0000996\;\;\;\times DP\right)\times e\left(0.03233\times DP-0.0000275\times DP^{2}\right)\right]/1000 \end{array}$$

where CBW is calf birth BW, kg, and DP is days pregnant.

### Statistical analysis

A Monte Carlo power analysis was conducted prior to the initiation of year 1, indicating that 8 cow/calf pairs per pen and 5 feeding levels (pens) replicated over year achieved 80% power in this controlled regression design. Pen was the experimental unit. Data was analyzed using the GLIMMIX procedure of SAS (SAS Inst. Inc., Cary, NC) as a linear mixed model. The most parsimonious model was selected based on the Akaike information criterion. The model included the linear and quadratic effects of maternal MEI as a fixed effect and the year of the experiment as a random effect. If the quadratic term was not significant, it was removed from the model. Significance of the fixed effects was declared when *P* ≤ 0.05, while tendencies were declared when 0.05 < *P* ≤ 0.10. Conditional R^2^ was calculated as described by Nakagawa and Schielzeth (2013) to determine the proportion of variance in the dependent variable that was explained by maternal MEI.

## Results and Discussion

### BW, BCS, and energy partitioning in cows

**Figure 1.**
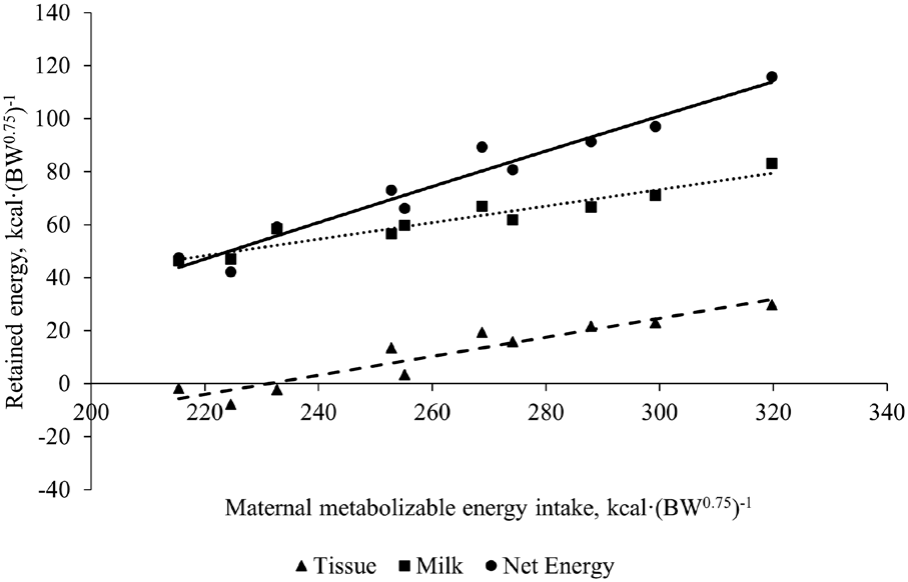
Tissue retained energy (triangles; Tissue = −88.04 + 0.37*x*, *P* < 0.001; *R*^2^ = 0.87), milk retained energy (squares; Milk = −20.55 + 0.31*x*, *P *< 0.001; *R*^2^ = 0.90) and total net energy retained (circles; Net Energy = −97.8 + 0.66*x*, *P *< 0.001; *R*^2^ = 0.93) across a range of daily maternal MEI in late-lactation Angus cows (*x*).

**Figure 2.**
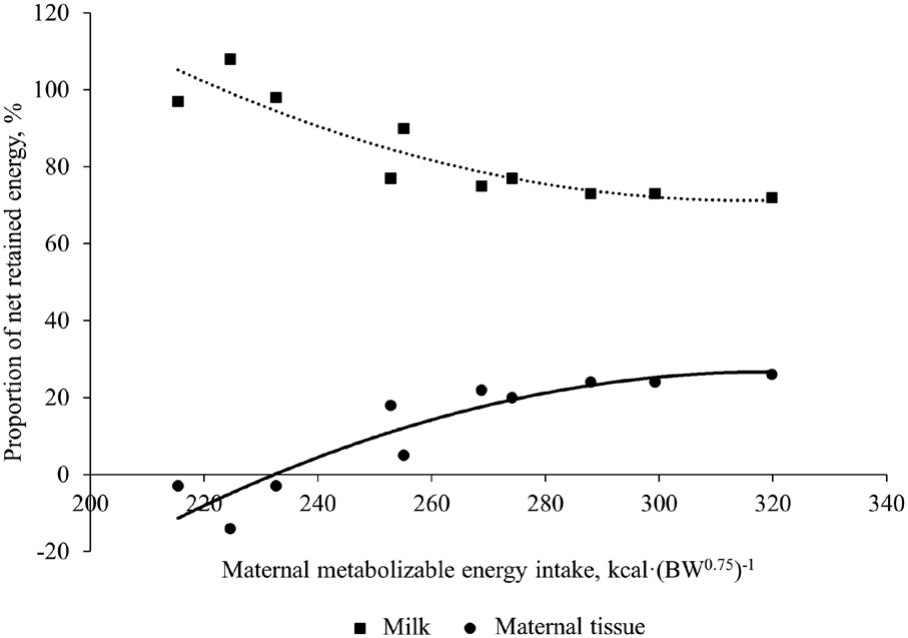
Relationship between maternal MEI (*x*) and proportion of net retained energy in milk (squares; Milk = 0.0028*x*^2^ − 1.852*x* + 376, *R*^2^ = 0.77) and maternal tissue (circles; Tissue = −0.003*x*^2^ + 1.939*x* − 297; *R*^2^ = 0.79) in mid- to late-lactation Angus cows.

Summary statistics for all measured parameters in cows, calves, and milk across year of experiment can be found in Table 3. Cows weighed less and had lower BCS at study initiation in year 2 compared to year 1. This was likely the cause of lower milk yield and milk nutrient concentration in year 2. However, calf ADG was similar across years. Cow BW and BCS adjusted to 100 d of experiment increased (*P *< 0.01, Table 4) 0.84 kg and 0.02 units, respectively, per unit increase in daily MEI kcal·BW^0.75^·d^−1^. Similarly, 100-d ADG increased by 0.0045 kg per unit increase in daily MEI. As expected, cow REA, IMF, rib fat, and rump fat increased (*P *< 0.01) with increasing MEI (Table 4). Figure 1 shows the relationship between MEI and retained energy in cows. Each additional kcal·(BW^0.75^)^−1^ MEI yielded 0.68 kcal·(BW^0.75^)^−1^ increase (*P *< 0.001) in total net energy retained. This partial efficiency coefficient is the same (0.68) derived from the equation of Garrett (1980) and is similar to that reported by Freetly et al., (2006; 0.69) and  Reynolds and Tyrell (2000; 0.64) using primiparous cows. Zero net energy retained, or maintenance energy requirement, occurred at 147.5 kcal MEI·(BW^0.75^)^−1^. The lactating cow default requirement used in the Beef Cattle Nutrient Requirements Model (NASEM, 2016) is 92.5 kcal·(BW^0.75^)^−1^ of net energy for maintenance (**NE**_**m**_). Therefore, using the partial efficiency coefficient from Garrett (1980), the NASEM (2016) default ME requirement for lactating beef cow maintenance is 136 kcal·(BW^0.75^)^−1^. Dividing the NASEM (2016) NE_m_ requirement for lactating cows by the partial efficiency coefficient derived from the current experiment yields a similar estimated maintenance requirement of 140 kcal ME·(BW^0.75^)^−1^. Retained energy is partitioned to NE_t_, NE_l_, and NE_y_ (NASEM, 2016). We estimated energy partitioned to NE_y_ averaged 4% of total net energy retained and was minimal due to the early stage of pregnancy cows were in throughout this experiment. Maternal tissue retained energy increased linearly [*P *< 0.001; 0.37 kcal·(BW^0.75^)^−1^ per unit increase in MEI]. Similarly, NE_l_ increased linearly (*P *< 0.001) by 0.31 kcal·(BW^0.75^)^−1^ per unit increase in MEI. Thus, the efficiency of MEI for maternal tissue accretion was 18.6% greater than efficiency of MEI used for milk energy production. This difference in partial efficiency is in agreement with NASEM (2021) where efficiency of accretion of tissue energy in dairy cows was assumed to be 12.2% greater than efficiency for milk energy production using the data from Beltsville Energy Metabolism Unit (Moraes et al., 2015), values for NRC (2001) and NASEM (2021). Figure 2 shows the relationship between maternal MEI, and the proportion of net retained energy partitioned to NE_t_ and NE_l_. As MEI increases, the proportion of energy recovered as NE_t_ rises at a decreasing rate. At approximately 275 kcal·BW^0.75^·d^−1^ of MEI, the proportion plateaus. The proportion of energy recovered as NE_l_ shows an inverse relationship with NE_t_. As MEI increases, a greater proportion of available net energy is diverted towards NE_t_, reaching a maximum proportion of 26%, with cows losing weight when subjected to the lower levels of MEI (between 215 and 233 kcal·BW^0.75^·d^−1^). In dairy cows, as lactation proceeds, insulin concentration and sensitivity of tissues increase, therefore, increasing glucose supply beyond that required for milk production increases plasma concentrations of glucose and insulin and partitioning of energy to body reserves (NASEM, 2021). Under these environmental conditions and the genetic capacity for milk yield in this cow herd, one should expect at least 74% of available net energy to be recovered as NE_l_. Conversely, at most 26% of available net energy should be recovered as NE_t_, during mid to late lactation.

**Table 4.** Regression models characterizing the effect of maternal MEI^1^ on body weight gain, and body composition of beef cows and their calves

Item	Intercept (SE)^2^		Linear coefficient (SE)		Quadratic coefficient (SE)		*P*-value			R-sq^3^
							Intercept	Linear	Quadratic	
Cow										
100-d BW^4^, kg	327	(50.2)	0.84	(0.190)			0.10	<0.01		0.69
100-d BCS^5^	−0.19	(0.954)	0.02	(0.003)			0.88	<0.01		0.59
100-d ADG^6^, kg·d^−1^	−0.85	(0.187)	0.0045	(0.00071)			0.14	<0.01		0.82
REA^7^, cm^2^	28.7	(7.81)	0.139	(0.0295)			0.17	<0.01		0.71
IMF^8^, %	1.9	(0.30)	0.008	(0.0011)			0.10	<0.01		0.75
Rib fat, cm	−0.47	(0.124)	0.0030	(0.00045)			0.16	<0.01		0.71
Rump fat, cm	−0.70	(0.196)	0.0040	(0.00073)			0.17	<0.01		0.70
Calf										
DMI, kg·d^−1^^9^	5.2	(0.500)	−0.004	(0.0019)			0.06	0.08		0.31
100-d BW, kg	223	(17.8)	0.185	(0.0671)			0.05	0.03		0.46
100-d ADG, kg	0.95	(0.07)	0.0017	(0.00020)			0.05	<0.01		0.39
REA, cm^2^	58.1	(5.11)	0.028	(0.0187)			0.06	0.17		0.13
IMF, %	9.1	(3.13)	−0.045	(0.0238)	0.0001	(0.00005)	0.21	0.11	0.10	0.36
Back fat, cm	0.38	(0.108)	0.00055	(0.000406)			0.17	0.22		0.17
Rump fat, cm	0.32	(0.114)	0.00097	(0.000416)			0.22	0.05		0.24

^1^Metabolizable energy intake = [ME of the feed (kcal) × DMI (kg)] / BW^0.75^ (kg) was calculated from regressing in vivo digestible energy values on DMI (g/kg BW^0.75^). Feed digestible energy was converted to metabolizable energy by multiplying by 0.82 (NASEM, 2016).

^2^Standard errors (SE) are shown in parentheses.

^3^Calculations of *R*^2^ based on Nakagawa and Schielzeth (2013).

^4^Body weight predicted at day 100 of experiment.

^5^Body condition score predicted at day 100 of experiment.

^6^100-d average daily gain.

^7^Rib eye area.

^8^Intramuscular fat.

^9^Calf daily feed dry matter intake.

### BW, ultrasound body composition, and energy efficiency in calves

Increasing maternal MEI did not affect REA (*P *= 0.17) nor back fat (*P *= 0.22) although rump fat increased slightly (linear *P *= 0.05) at the rate of 0.1 cm per 100 kcal maternal MEI·BW^0.75^·d^−1^. Percent IMF tended (*P *= 0.10) to increase in a curvilinear fashion when the dam received > 230 kcal·BW^0.75^·d^−1^. This change was minimal with an increase of only 0.5 percentage units when maternal MEI was increased from 200 to 300 kcal·BW^0.75^·d^−1^ (4.1 to 4.6 % IMF, respectively). Calf BW adjusted to 100 d of experiment linearly increased (*P *= 0.03) by 0.185 kg per unit increase in maternal MEI, with ADG showing a similar trend (*P *< 0.01).

**Figure 3.**
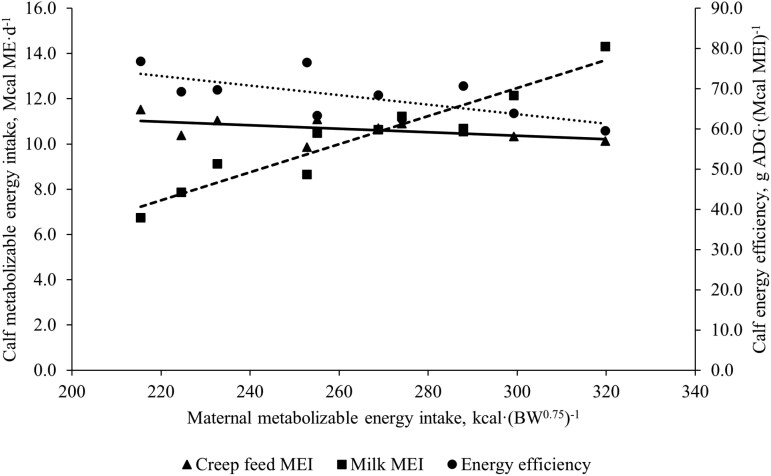
Daily MEI from creep feed (triangles; Creep feed MEI = 12.69 − 0.008*x*, *P* = 0.12; *R*^2^ = 0.25), milk (squares; Milk MEI = −5.55 + 0.060*x*, *P* < 0.001; *R*^2^ = 0.88) and energy efficiency (circles; Calf energy efficiency, calculated as g of ADG per Mcal of MEI; Calf energy efficiency = 89.51 − 0.082*x*, *P* = 0.03; *R*^2^ = 0.20) in Angus calves suckling mid to late-lactation Angus cows across a range of maternal MEI (*x*).

Figure 3 shows the relationship between maternal MEI (kcal·(BW^0.75^)^−1^) and calf MEI (kcal ME·d^−1^) from milk and creep feed, as well as energy efficiency of calves. Increasing maternal MEI linearly increased (*P *< 0.001) milk energy availability and therefore, we assumed calf energy intake from milk increased accordingly. Neither creep feed intake nor creep feed digestibility (data not shown) were affected by level of maternal MEI (*P ≥* 0.12). Results from the literature evaluating the relationship of dam’s milk production to progeny dry feed or forage intake and digestibility are mixed. In beef production systems, most reports reflect a preference for calves to consume all the milk available regardless of the presence of creep feed or forage for grazing (Baker et al., 1976; Buskirk et al., 1992; Abdelsamei et al., 2005; Tedeschi and Fox, 2009). In experiments where creep feed was offered to calves on an ad libitum basis, feed energy intake of the calves was not different (Green et al., 1991; Jenkins et al., 1991) or increased (Bowden 1980; Brown and Dinkel, 1982) with declining milk production of the dam. Abdelsamei et al. (2005) supplied 5 levels of peak milk production along with ad libitum alfalfa hay to Holstein steer calves. The results indicated a 43% decrease in forage consumption with a 22% increase in milk intake between the highest and lowest milk intake treatment groups. Similar to our results, Abdelsamei et al. (2005) did not find a difference in DM digestibility with increased milk intake.

The overall energy efficiency of the calves was linearly and negatively affected (*P *= 0.03) by increasing maternal MEI. Efficiency of calf growth (g ADG·(Mcal MEI)^−1^) decreased by 11.5% when maternal energy intake was increased by 100 kcal·BW^0.75^·d^−1^. This response occurred even though per100 kcal increase in maternal MEI, there was a 78% increase in milk MEI, and a 12.8% increase in 100-d calf ADG. Overall, the results suggest that the potential benefit of increasing maternal MEI on milk energy available for the calf (6 Mcal ∙ d^−1^) is offset by a declining efficiency of ME used for calf growth.

### Milk yield and composition

Cows in the current experiment produced an average of 8.5 to 9.5 kg of milk per day (year 1 and year 2, respectively) compared to 6.4 to 8.6 kg of milk production per day in previous literature for this stage of lactation and using similar methods (Miller et al., 1999; Winterholler et al., 2012). Milk yield linearly increased (*P *< 0.01) with increased MEI (Table 5), suggesting that maximum observed milk yield was limited by MEI and not by genetic capacity. Similarly, Moe et al. (1965) reported that with increasing energy availability, a greater portion of the diet is available to support milk production in dairy cows. Breed by maternal MEI interactions have been reported by Jenkins and Ferrell (1992) and Jenkins et al. (2000) , where breeds with greater genetic capacity for milk yield responded positively and linearly to increasing MEI. However, breeds with low genetic capacity did not respond by increasing milk yield as MEI increased from 170 to 290 kcal·BW^0.75^·d^−1^ (Jenkins and Ferrell, 1992). Interestingly, Angus-sired cows in the current experiment produced 0.04 kg more milk yield whereas Angus-sired cows in the experiment of Jenkins and Ferrell (1992) produced only 0.0134 kg more milk yield for each kcal·BW^0.75^·d^−1^ increase in MEI, respectively. However, in the study of Jenkins and Ferrell (1992) MEI was estimated using book values for feed energy concentration; diet digestibility was not measured. Therefore, we adjusted MEI intake in their experiment using the relationship of in vivo MEI to calculate MEI from the current experiment, as follows:

**Table 5.** Regression models depicting the effect of maternal MEI^1^ on mid- and late-lactation milk production and milk composition

Item	Intercept (SE)^2^		Linear coefficient (SE)		*P*-value		*R* ^2^ ^3^
					Intercept	Linear	
Milk yield, kg. d^−1^	−2.1	(1.79)	0.04	(0.007)	0.45	<0.01	0.81
Fat, %	2.25	(0.645)	0.005	(0.0020)	0.18	0.04	0.08
Protein, %	2.38	(0.188)	0.003	(0.0007)	0.05	0.01	0.58
Lactose, %	4.51	(0.137)	0.001	(0.0005)	0.02	0.04	0.43
SNF^4^, %	7.81	(0.226)	0.004	(0.0008)	0.02	<0.01	0.51
MUN^5^, %	20.9	(3.27)	−0.019	(0.0041)	0.10	<0.01	0.02
Milk energy^6^, Mcal/kg	0.54	(0.066)	0.001	(0.0002)	0.08	0.01	0.20

^1^Metabolizable energy intake (*x* axis) = [ME of the feed (kcal) × DMI (kg)] / BW^0.75^ (kg) was calculated from regressing in vivo digestible energy values on DMI (g/kg BW^0.75^). Feed digestible energy was converted to metabolizable energy by multiplying by 0.82 (NASEM, 2016).

^2^Standard errors (SE) are shown in parentheses.

^3^Calculations of *R*^2^ based on Nakagawa and Schielzeth (2013).

^4^Solids nonfat.

^5^Milk urea nitrogen.

^6^Milk energy concentration, Mcal NE_l_ ∙ kg milk = (0.092×%MkFat) + (0.049×%MkSNF)‒0.0569, where %MkFat is concentration of fat and %MkSNF is concentration of solids nonfat.


$$\begin{array}{l} MEI=75.66+0.721\;\times MEI_{c} \end{array}$$

where MEI is maternal MEI in kcal adjusted for feed intake and MEI_c_ is kcal of MEI calculated using feed intake and book values for feed energy concentration. After adjusting for diet digestibility, the milk yield response to increasing maternal MEI in the study of Jenkins and Ferrell (1992) was estimated to be 0.0185 kg per kcal·BW^0.75^·d^−1^ increase in MEI and thus substantially lower than the response observed in the current experiment. Increased response to MEI is likely due to aggressive selection over time for maternal influence on calf weaning weights, and thus increased genetic capacity for milk yield.

In dairy cows, some authors reported a quadratic response for energy corrected milk (**ECM**) yield to increasing levels of energy intake in multiparous cows while the increment seems to be more linear in primiparous cows (Jensen et al., 2015; Daniel et al., 2016). Few studies are available to determine the influence of dietary energy on milk composition in beef cows. Except for milk fat concentration, mean milk composition values in the current study were slightly lower than those presented in previous literature (Lalman et al., 2000; Hudson et al., 2010; Winterholler et al., 2012; Rodrigues et al., 2014; NASEM, 2016; Edwards et al., 2017). Concentrations of nutrients in milk increased linearly (*P *≤ 0.04) with increasing MEI, except in the case of milk urea nitrogen that was negatively affected (*P *< 0.01). Overall, milk energy production linearly increased (*P *= 0.01) with increasing MEI. In dairy cows, some authors reported that primiparous and multiparous dairy cows in early and mid-lactation had a milk energy yield of 0.002 Mcal/kg of milk per kcal·BW^0.75^·d^−1^ of MEI (Jensen et al., 2015), while others reported a slightly greater milk energy of 0.003 Mcal /kg of milk per kcal·BW^0.75^·d^−1^ of MEI in multiparous dairy cows (Daniel et al., 2016). In dairy cows, an increased supply of NE_l_ above maintenance requirements is expected to increase milk energy yield (0.002 Mcal/kg of milk per additional kcal of NE_l_), due to a greater daily yield of fat, protein and lactose, although this increased supply of NE_l_ has also been associated with lower concentration of fat in milk (Daniel et al., 2016). It has been reported that using NE_l_ intake rather than DMI in regression models improved prediction of ECM response in dairy cows (Jensen et al., 2015). In their work, ECM response was greater in multiparous cows than primiparous cows (Jensen et al., 2015). Using primiparous beef cows, Lalman et al. (2000) also found similar responses in which milk protein increased linearly and milk fat increased in a curvilinear fashion with increasing levels of energy supplied. Additional work from Friggens et al. (1995) reported an increase in milk fat content with increasing dietary energy in Friesian dairy cows. Several reviews have indicated that unlike milk fat and milk protein, lactose concentration is rarely altered with dietary changes in dairy cows (Sutton, 1989; Sutton and Morant, 1989). However, Broderick (2003) demonstrated increasing lactose concentrations with increasing dietary energy in dairy cattle. Daily milk energy production and milk nutrient composition is highly sensitive to the amount of feed energy available to beef cows with similar genetic potential and managed under similar conditions.

## Conclusions

As maternal MEI increased, milk energy yield and maternal tissue energy accretion increased simultaneously and in a linear fashion. Although, the increasing availability of MEI to the dam increased MEI in the form of milk to the calf, calf efficiency of energy utilization decreased. Given similar conditions and genetic potential for milk production, these data can be used to estimate the influence of energy supply on milk composition, milk energy production and (or) energy required to achieve predetermined targets in cow BCS. More work is needed to characterize these relationships during early lactation and given a wider range in both maternal MEI and genetic capacity for milk yield.

## Abbreviations

ADIA: acid detergent insoluble ash
ADF: acid detergent fiber
ADG: average daily gain
AI: artificial insemination
AIC: Akaike Information Criterion
BW: body weight
BW^0.75^: metabolic body weight
BCS: body condition score
CBW: calf birth body weight
CIDR: controlled internal drug release
CP: crude protein
DE: digestible energy
DM: dry matter
DMI: dry matter intake
DP: days pregnant
ECM: energy corrected milk
EE: ether extract
IACUC: Institutional Animal and Care Use Committee
IM: intramuscularly
IMF: intramuscular fat
ME: metabolizable energy
MEI: maternal metabolizable energy intake
MEI_c_: metabolizable energy intake calculated using feed intake and book values for feed energy concentration
MEP: milk metabolizable energy production
MP: metabolizable protein
MUN: milk urea nitrogen
NDF: neutral detergent fiber
NE_m_: net energy for maintenance
NE_l_: net energy of lactation
NEr: total net energy retained
NE_t_: maternal tissue retained energy
NE_y_: net energy required for pregnancy
RDP: rumen degradable protein
REA: rib eye area
SNF: solids nonfat
TMR: total mixed ration
TDN: total digestible nutrients
